# Atrial Flutter Mechanism Detection Using Directed Network Mapping

**DOI:** 10.3389/fphys.2021.749635

**Published:** 2021-10-26

**Authors:** Muhamed Vila, Massimo Walter Rivolta, Giorgio Luongo, Laura Anna Unger, Armin Luik, Lorenzo Gigli, Federico Lombardi, Axel Loewe, Roberto Sassi

**Affiliations:** ^1^Dipartimento di Informatica, Università degli Studi di Milano, Milan, Italy; ^2^Institute of Biomedical Engineering, Karlsruhe Institute of Technology, Karlsruhe, Germany; ^3^Medizinische Klinik IV, Städtisches Klinikum Karlsruhe, Karlsruhe, Germany; ^4^UOC Malattie Cardiovascolari, Fondazione IRCCS Ca' Granda Ospedale Maggiore Policlinico, Milan, Italy

**Keywords:** cardiac arrhythmias, network theory (graphs), atrial flutter, electrograms, catheter ablation

## Abstract

Atrial flutter (AFL) is a common atrial arrhythmia typically characterized by electrical activity propagating around specific anatomical regions. It is usually treated with catheter ablation. However, the identification of rotational activities is not straightforward, and requires an intense effort during the first phase of the electrophysiological (EP) study, i.e., the mapping phase, in which an anatomical 3D model is built and electrograms (EGMs) are recorded. In this study, we modeled the electrical propagation pattern of AFL (measured during mapping) using network theory (NT), a well-known field of research from the computer science domain. The main advantage of NT is the large number of available algorithms that can efficiently analyze the network. Using directed network mapping, we employed a cycle-finding algorithm to detect all cycles in the network, resembling the main propagation pattern of AFL. The method was tested on two subjects in sinus rhythm, six in an experimental model of *in-silico* simulations, and 10 subjects diagnosed with AFL who underwent a catheter ablation. The algorithm correctly detected the electrical propagation of both sinus rhythm cases and *in-silico* simulations. Regarding the AFL cases, arrhythmia mechanisms were either totally or partially identified in most of the cases (8 out of 10), i.e., cycles around the mitral valve, tricuspid valve and figure-of-eight reentries. The other two cases presented a poor mapping quality or a major complexity related to previous ablations, large areas of fibrotic tissue, etc. Directed network mapping represents an innovative tool that showed promising results in identifying AFL mechanisms in an automatic fashion. Further investigations are needed to assess the reliability of the method in different clinical scenarios.

## 1. Introduction

Atrial arrhythmias include many diverse rhythm disturbances for which a wide range of different arrhythmia mechanisms are responsible. Generally, these arrhythmias respond poorly to antiarrhythmic drugs and after many years of technological advances in cardiac electrophysiology, catheter ablation is today recognized as the treatment of choice (Lee et al., 2012).

Atrial flutter (AFL) is a common supraventricular arrhythmia characterized by a reentrant circuit around a central obstacle, which can be a fixed anatomical structure or a functional electrophysiological line of block (Cosio et al., 2006). Depending on the obstacle, AFL is usually classified as “typical”, if the re-entry is around the tricuspid valve, or “atypical” if the tricuspid valve is not involved (Saoudi et al., 2001; Bun et al., 2015). Atypical AFL is often associated with structural heart disease, especially in patients that have undergone cardiac surgery or extensive catheter ablation for the treatment of atrial fibrillation (AF). In these cases, an electrophysiological (EP) study is the most common way to unveil the mechanisms causing the arrhythmia and plan a proper ablation (Gerstenfeld et al., 2004). Although AFL is not directly related to death, it affects life quality due to the higher ventricular activation rate, and it can cause significant complications such as heart failure and stroke (Biblo et al., 2001). Also, the presence of AFL usually suggests an underlying predisposition to AF, which is a more complex arrhythmia (Waldo and Feld, 2008).

Ablation approaches guided by activation maps obtained from three-dimensional (3D) electro-anatomical mapping systems try to localize and target atrial arrhythmia drivers. Computerized mapping systems are of crucial importance for delineating the more complex circuits of atypical AFL (particularly in the context of abnormal atrial anatomy, multiple circuits, and regions with scars). Successful ablation is dependent on identification of a critical isthmus in the reentrant circuit, which can be interrupted either with a line, or focal point of ablation (Lee et al., 2012). If an incorrect target is ablated, the patient will certainly not be cured and, due to scarring with an ineffective ablation procedure, there is an additional risk that a new arrhythmia may be induced (Chugh et al., 2005; Deisenhofer et al., 2006). Moreover, there are indications that AFL and AF could be expressions of a single arrhythmogenic substrate, which would make it significant to fine-tune the concept of “curing” AFL (Cosio et al., 2006). With the aim of optimizing catheter ablation, we believe new tools for the assessment of cardiac excitation patterns are needed to help determine the underlying mechanisms, and to improve the identification of the appropriate targets.

In spite the considerable variety of network theory applications in many disciplines, only recently directed networks have been applied with a goal to identify the sources of cardiac arrhythmias. Zahid et al. (2016) proposed to use the “minimum cut” algorithm based on network flow analysis to predict optimal ablation targets for left AFL. Vandersickel et al. (2019) demonstrated the wide applicability of directed networks for the detection of driving mechanisms of cardiac arrhythmias (focal or reentrant) in both the atria and ventricles. The same authors applied the method to more complex clinical atrial tachycardia cases (Van Nieuwenhuyse et al., 2021b). This latter work is very recent and resembles similarities with our work, which we discuss in the section 4.

In this study, we formally define a technique, that we called “directed network mapping”, recently introduced in our preliminary work (Vila et al., 2021) and inspired by the work of Vandersickel et al. (2019). Directed network mapping creates a directed network by processing intracardiac electrograms (EGMs) to model the electrical propagation on the atrial surface. Atrial conduction paths can be indeed identified based on the time delay between activations collected at two locations at close distance. Then, network theory algorithms can automatically identify arrhythmia mechanisms from the network created. The goal of this study is to verify the applicability of directed network mapping for the identification of AFL mechanisms in both *in-silico* and clinical settings. We tested the accuracy of directed network mapping in 10 simulated atrial models of various AFL types and in 10 clinical AFL cases.

## 2. Materials and Methods

### 2.1. Creation of a Directed Network for Electrical Mapping

**Figure 1:**
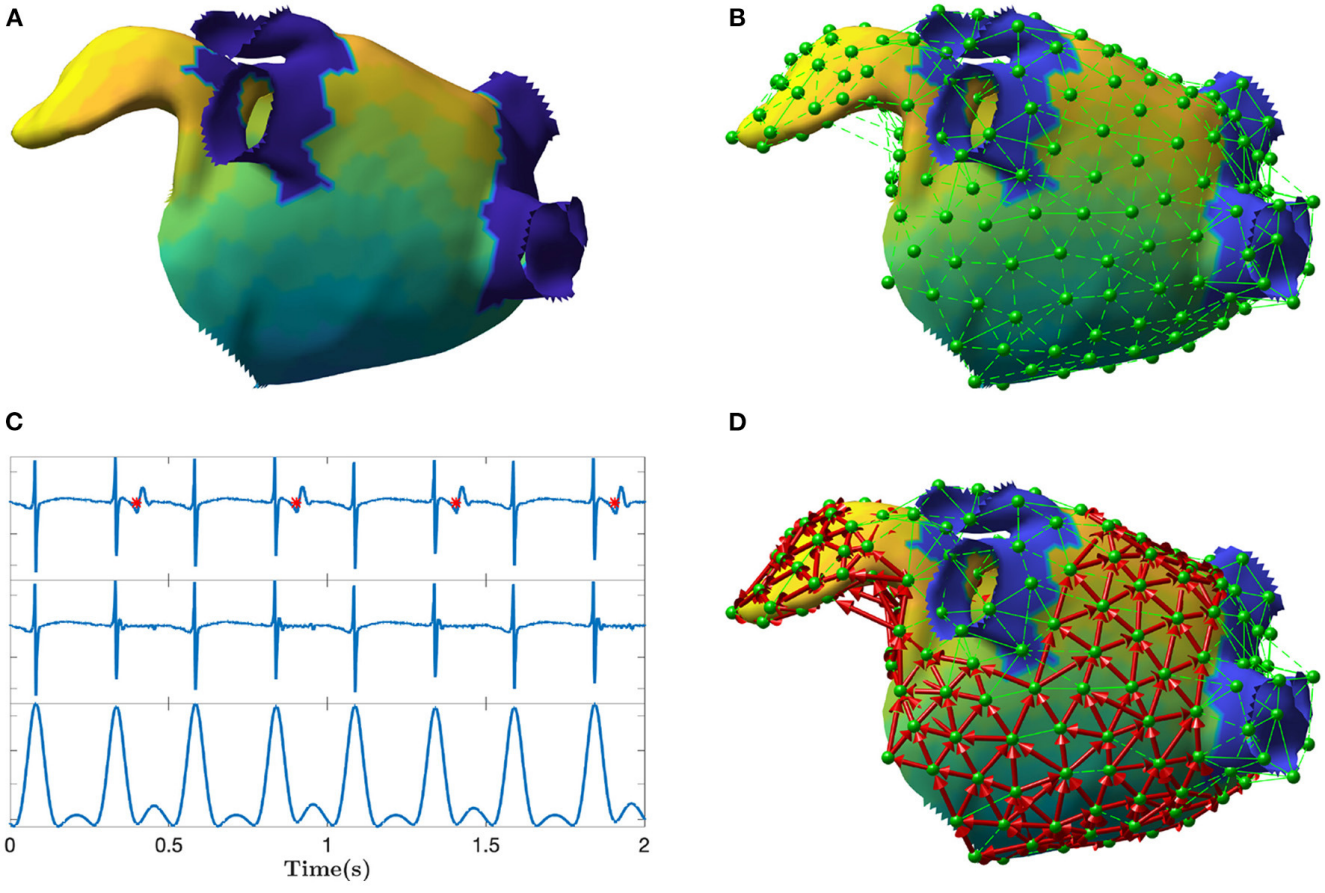
Depiction of the main steps of the directed network mapping technique. **(A)** Left atrial 3D model with LAT colormap. **(B)** Set of nodes (in green) equally distributed on the atrial surface with neighboring nodes connected using Delaunay triangulation. **(C)** Ventricular activity cancellation and preprocessing of one unipolar recording. The top panel of **(C)** shows original EGM and the time instants of the ventricular activity are marked in red. The middle panel shows the same EGM after performed cancellation. The bottom panel shows the result of the EGM filtering meant to emphasize the activations. The amplitude scale is in arbitrary units. **(D)** Final directed network (in red) after processing all the signals at each node to determine the existence and direction of the connections between nodes. The color map represents the LAT value across the atrial surface referenced to the activation of a specific node in the mesh.

In this work, electrical propagation on the atrial surface was modeled using network theory. In general, a network is composed of nodes which are connected through edges. In our context, nodes were defined as specific sites on the atrial surface and edges represented the directed electrical propagation from one site to a nearby one, which made the network a “directed” one. In this section, we proposed an algorithm to build the directed network from the unipolar electrograms (EGM) and electrode coordinates (spatial positions) acquired during sequential mapping in an EP study. That is, we linked the electrical activation of the cells in the atria with a corresponding network made of *M* nodes. The main steps of the algorithm are illustrated in Figure 1.

The algorithm started selecting a set of *M* network nodes equally distributed (as much as the geometry permits) on the entire atrial endocardial surface. The *i*-th node was defined by its spatial 3D position **p**_*i*_ = [*x*_*i*_, *y*_*i*_, *z*_*i*_]. In addition, for each node *i*, we expected its corresponding unipolar EGM **Φ**_*i*_, collected at that spatial position **p**_*i*_. **Φ**_*i*_ was the vector of the EGM samples.

The first step of the algorithm was the preprocessing of each EGM. The major disadvantage of atrial unipolar recordings is the contamination of substantial far-field ventricular activity, that typically overlaps the signal of interest. For this reason, ventricular activity cancellation was performed. Many cancellation techniques exist in the literature (Sörnmo, 2018). We used an advanced cancellation strategy, which integrates local modeling of the atrial activity and average beat subtraction (Rivolta et al., 2019). Figure 1C exemplifies the cancellation process on a single unipolar recording.

In order to model wave propagation into the network, only nodes spatially close to each other on the atrial surface were allowed to be connected through an edge. Delaunay triangulation technique was applied to define the set of neighbors for each node *i*, hereafter mathematically defined as $B_{i}$. All nodes connected to the node *i* through a triangle were considered as neighbors. An example of neighbors determination is illustrated in Figure 1B.

The next step was to establish whether an edge from node *i* to any of its neighbors in $B_{i}$ had to be created based on the EGM at the nodes. We created a connection only if the conduction velocity, estimated for the electrical wave propagating between node *i* and $j\in B_{i}$, was within a physiological range. In other words, only electrical waves propagating in certain directions with respect to the vector connecting the nodes *i* and *j* were eligible to determine a connection. The conduction velocity criterion was defined as follows


(1) $$\begin{array}{l} {\text{CV}}_{\text{min}}<{\text{CV}}_{ij}=\frac{d_{ij}}{\tau_{ij}}<{\text{CV}}_{\text{max}} \end{array}$$

where CV_min_ and CV_max_ were set to 10 and 250 cm/s, according to physiological limits (Konings et al., 1994; Harrild and Henriquez, 2000); *d*_*ij*_ was the Euclidean distance between **p**_*i*_ and **p**_*j*_ in cm, and τ_*ij*_ was the time delay between **Φ**_*i*_ and **Φ**_*j*_ in seconds. The delay τ_*ij*_ was estimated using the cross-correlation function between **Φ**_*i*_ and **Φ**_*j*_. In particular, τ_*ij*_ was set as the time delay associated to the first maximum of the cross-correlation between the two signals. To reduce the effect of noise and spurious local morphology, before computing the cross-correlation, **x**_*i*_ and **x**_*j*_ were preprocessed with bandpass filtering (3rd order Butterworth, 40–250 Hz, zero phase), rectification, and lowpass filtering (3rd order Butterworth, 20 Hz, zero phase) (Botteron and Smith, 1995). Figure 1C illustrates how the preprocessing emphasized the activations in the signals as opposed to signal morphology. In case the delay τ_*ij*_ was negative and the absolute value of CV_*ij*_ was within the physiological range, the directed edge was created from node *j* to node *i* (instead of *i* to *j*). As common in network theory, the connections between the nodes in the network are described using a connectivity matrix **C** with entries *c*_*ij*_. The existence of a directed edge between nodes *i* and *j* was set by having *c*_*ij*_ = 1, and 0 otherwise.

In order to compute the time delay τ_*ij*_ using cross-correlation, the EMGs **Φ**_*i*_ and **Φ**_*j*_ had to contain at least one local atrial activation each. For each time window of length *l*, a network was created using the approach described so far. In addition, in order to detect sustained atrial arrhythmia, the creation of the network was repeated on consecutive windows (with possible overlap) and then were “averaged” to build a final directed network **A**, whose entries were defined as follows:


(2) $$\begin{array}{l} a_{ij}=\{\begin{array}{ll} 1 & \frac{1}{N}\sum_{n=1}^{N}c_{ij}^{n}\geq \frac{1}{N}\sum_{n=1}^{N}c_{ji}^{n}+\gamma \\ 0 & \text{otherwise} \end{array} \end{array}$$

where *N* was the total number of networks “averaged”, $c_{ij}^{n}$ the entry in the connectivity matrix **C**_*n*_ for the *n*-th network, and γ ≥ 0 a threshold parameter. A connection in **A** was established from node *i* to node *j*, setting *a*_*ij*_ = 1, if the average connection strength $\sum_{n=1}^{N}c_{ij}^{n}/N$, in direction *i* to *j*, was larger than the average connection $\sum_{n=1}^{N}c_{ji}^{n}/N$, in the opposite direction, *j* to *i*, plus a small positive safety threshold γ, used to avoid random connection, due to noise, to appear.

### 2.2. Detection of Cycles in the Directed Network

Having at disposal the directed network corresponding to an electrical mapping, macroreentries can be found by detecting cycles (i.e., closed-loops) in the network. A cycle L is defined as a non-empty sequence of nodes linked by directed edges in which the first and last node coincide. A standard depth-first search (DFS) algorithm was used for the purpose of detecting the cycles.

Given the large number of nodes in the network, in real applications, the cycle finding algorithm may provide cycles that are very similar to each other, and differ only by a few nodes (in many cases, just one). These cycles belong to the same anatomical region and, in practical terms, should be considered as a single one. A network-based grouping algorithm of these cycles, based on the amount of nodes shared, was designed by means of the following three steps.

First, the amount of shared nodes between cycle *h* and *k* was computed as follows


(3) $$\begin{array}{l} w_{hk}=\frac{\#L_{h}⋂L_{k}}{\text{sup}\left\{\#L_{h},\#L_{k}\right\}} \end{array}$$

where # was the cardinality of a set, $\#L_{h}⋂L_{k}$ represented the number of nodes shared between $L_{h}$ and $L_{k}$, that were then normalized by the length of the longest cycle. Hence, the quantity *w*_*hk*_ was bounded, by construction, between 0 and 1.

Second, each *w*_*hk*_ was used to build a further undirected graph whose nodes were cycles. A connectivity matrix **W** was built using the values of *w*_*hk*_. In the graph, the edge between cycle *h* and *k* existed only if *w*_*hk*_ was greater than a certain threshold *t*. Intuitively, a low value of *t* facilitates the merging of the cycles. In our study, *t* was empirically set to 0.6 by visual inspection of the grouping algorithm results.

Third, a standard algorithm based on DFS was applied to locate connected components in the undirected graph just created. A connected component of an undirected graph is a set of nodes such that each pair is connected by a path. All the cycles belonging to the same connected component were grouped together and considered to represent the same reentry.

### 2.3. Directed Network Mapping for Sequential Data

In the clinical setting, ablation procedures are guided by the results of electro-anatomical mapping. Data are derived from recordings of multipolar catheters, which are moved inside the atria to map the relevant regions and guide the intervention. Several catheter configurations are available on the market, and usually present a few electrodes (in the range of a few tens). With this typical setting, only snapshots of a few seconds of EGMs in the current position of the electrodes become available. In order to map the electrical activity of both atria, sequential mapping is used to construct both voltage and activation maps. The latter is built by using a temporal reference, usually the QRS complex of the surface electrocardiogram (ECG) or the activation detected in a catheter inserted in the coronary sinus, to temporally align the activations identified within successive snapshots for each electrode. In addition, the positions of the electrodes are also tracked over time, with a magnetic- and impedance-based navigation system, thus allowing the construction of a 3D map of the geometry and of the electrical activation. In our study, we (retrospectively) utilized the data collected via sequential mapping to create a directed network that models the electrical propagation during AFL.

Sequential mapping brings two issues for directed network mapping. First, the number of atrial locations at which EGMs are collected is typically too large for building a directed network meant to model the electrical propagation. Many of these EGMs are acquired on locations very close between each other. However, current navigation systems report localization errors of about 1 mm (Jiang et al., 2009). Hence, the electrical propagation between very close electrodes cannot be estimated reliably. In addition, a large number of nodes prevents the network to be efficiently processed by the network theory algorithms, thus hampering the use of the technique during EP studies. Second, the recordings are not acquired concurrently at the same time (EGMs are collected sequentially with the same probe, which is moved).

In order to tackle these two issues, a coarsening procedure was designed to build a network composed of a smaller number of nodes *M*, each associated with an EGM temporally aligned with the others. We proceeded as follows. First, having at disposal a virtual anatomy (output of the electro-anatomical mapping during ablation), *M* points were distributed all over the geometric mesh of the atrial surface and the Delaunay triangulation technique was applied. The output of this step were the 3D coordinates **p**_*i*_ of each node, each contained in a triangular tessellation involving the neighboring ones. The next step was to assign the electrical activities measured by the moving catheter to each **Φ**_*i*_. The position and electrical activity of each electrode on the catheter were tracked over time. Let ${\tilde{p}}_{en}$ and ${\tilde{\Phi }}_{en}$ be the 3D position and electrical activity of electrode *e* at time index *n*, respectively. The assignment was performed by (i) checking whether the average position of the electrode was near to one of the nodes **p**_*i*_; and (ii) the 3D coordinates of the electrode did not vary in the window of observation (i.e., the maximum range of movement of the catheter across axes did not exceed a given threshold). The window was defined as *l*-samples-wide and centered on the reference QRS complex of each beat. More formally, let $W_{q}$ be the set of *l* time indices centered on the *q*-th QRS complex (where $\#W_{q}=l$, ∀*q*). The voltage samples ${\tilde{\Phi }}_{en}$, collected with the electrode *e* in the window of observation (i.e., $n\in W_{q}$), were assigned to the node *i* if the two following conditions were matched


(4) $$\begin{array}{r} \left\|{\text{p}}_{i}-\frac{1}{l}\underset{n\in W_{q}}{\sum }{\tilde{p}}_{en}\right\|<r_{1}\\ \underset{x,y,z}{\max}\left(\underset{n}{\max}{\tilde{p}}_{en}-\underset{n}{\min}{\tilde{p}}_{en}\right)<r_{2} \end{array}$$

where *r*_1_ and *r*_2_ were two thresholds set to assess the closeness to the node of the electrode and its position stability in time. They were empirically fixed at 6 and 2 mm, respectively. The max and min operators assessed the maximum and minimum values, along a given axis, of a vector or matrix, respectively.

This procedure may assign multiple signals to each node due to the presence of many QRS complexes and the many EGMs collected. In order to determine whether the directed edge should be created from node *i* to *j*, the delays between all pairs of signals associated with node *i* and *j* were computed using the procedure described in section 2.1, and a *t*-test was performed to verify if the average delay was significantly different from 0 ms (significance level α: 0.05). Then, if the difference was statistically significant, the edge was created only if the conduction velocity associated with the average delay was within the physiological range, as assessed by Equation (1).

### 2.4. Validation of Directed Network Mapping in Sinus Rhythm

Directed network mapping was first validated using data from two subjects in sinus rhythm (ages 67 and 54, both male). The data were collected from Fondazione I.R.C.C.S. Ospedale Maggiore Policlinico in Milan, Italy. Electro-anatomic mapping was performed using multielectrode catheters and the CARTO® 3 (Biosense Webster Inc., Irvine, CA, USA) navigation and mapping system. To allow correct local electrogram assessment, the default protocol mandated that the mapping catheter was maintained in each location for 2.5 s after points were acquired. Although patients were paroxysmal AF cases undergoing pulmonary vein (PV) isolation, they were in sinus rhythm during the mapping and had no prior known substrate modifications. The CARTO® 3 system provided activation maps in the form of local activation times (LAT). Every patient that underwent transcatheter cardiac mapping and ablation procedure had signed an informed consensus statement about periprocedural risk and about the use of clinical data for clinical research in the respect of privacy policy. A directed network map was built as described in the previous section and the results checked by visual inspection, comparing the connections of the network (connectivity matrix) with the LAT map.

### 2.5. Validation of Directed Network Mapping in Simulated AFL Cases

Next, we validated the directed network mapping algorithm in simulated AFL scenarios. To do so, based on simulated AFL mechanisms implemented in a previous work by Luongo et al. (2021), we retrospectively analyzed 6 different computational AFL scenarios. These simulations included right AFL as well as left AFL forms, like macroreentries around the valves and across the roof. A complete list of scenarios is provided in Table 1.

**Table 1:** List of simulated AFL mechanism scenarios.

**No**.	**Atrium**	**Mechanism**	**Position**	**Direction**
1	Right	Macroreentry	Tricuspid valve	CCW
2	Right	Macroreentry	Tricuspid valve	CW
3	Left	Macroreentry	Mitral valve	CW
4	Left	Figure-of-eight macroreentry	Both PVs	Anterior
5	Left	Figure-of-eight macroreentry	Both PVs	Posterior
6	Left	Figure-of-eight macroreentry	Right PVs	Anterior

Cardiac excitation was modeled using the fast marching approach to solve the Eikonal equation (Jacquemet, 2010; Trachtler et al., 2015). The atrial electrophysiological activity was simulated on the triangulated volumetric mesh of a bi-atrial anatomy (Figure 1A), generated from segmented magnetic resonance imaging data of a healthy subject (Krueger et al., 2013). Inter-atrial connections and fiber orientation were generated by a rule-based algorithm (Wachter et al., 2015; Loewe et al., 2016). Scars were added circumferentially around ipsilateral pulmonary veins representing ablation scars from the previous pulmonary vein isolation intervention. The simulations were initiated by manually placed triggers and refractory areas. They were continued at least 5 s to confirm a stable excitation pattern. The simulated excitation resulted in a LAT for each anatomical node that was not isolated. Spatio-temporal transmembrane voltage distributions were derived from the LATs using the Courtemanche human action potential model adapted to AF conditions (Loewe et al., 2016).

For each scenario, we distributed 400 equidistant nodes on the endocardial surface: half of them in the left and the other half in the right atrium (with 7.8 mm average distance between two neighboring nodes). The spatial distribution of nodes is visible in Figure 1B. The 3D coordinates of the nodes, along with corresponding synthetic unipolar EGMs, were used as input to the network mapping algorithm. The unipolar pseudo-EGM Φ_*i*_ for the *i*-th node was calculated using the infinite volume conductor approximation:


(5) $$\begin{array}{r} \Phi_{i}\left(x,y,z\right)=-\frac{1}{4\pi }\frac{\sigma_{\text{intra}}}{\sigma_{\text{extra}}}\int \int \int \nabla V_{m}\left(x^{\prime },y^{\prime },z^{\prime }\right)\\ \cdot \nabla \left(\frac{1}{r}\right)\;dx^{\prime }dy^{\prime }dz^{\prime } \end{array}$$

where σ_intra_ is the intracellular conductivity (within the cardiac tissue), σ_extra_ the extracellular conductivity (within the whole domain), $\nabla V_{m}\left(x^{\prime },y^{\prime },z^{\prime }\right)$ is the spatial gradient of the transmembrane voltage in the point with coordinates *x*′, *y*′, *z*′ (Malmivuo and Plonsey, 1995). The values of Φ_*i*_(*x, y, z*) computed over time were then stored in the vector **Φ**_*i*_.

The results were validated by locating the cycles in the network (section 2.2) and visually comparing their locations with the LAT map produced by the simulated mechanism.

### 2.6. Validation of Directed Network Mapping in Clinical AFL Cases

The algorithm was retrospectively validated on 10 patients (age: 66 ± 5 years; male/female: 7/3) with AFL who underwent an EP study and radiofrequency catheter ablation. The subjects selected by the clinicians were all complex AFL cases who had a history of AF and at least a previous pulmonary vein isolation or additional substrate modifications. The study was done using a 64 mini-electrode small basket array (IntellaMap Orion™, Boston Scientific, Inc., Malborough, USA) that enabled rapid high-density mapping. The cases were provided by Stdtisches Klinikum Karlsruhe, Germany and included 2 AFL scenarios in the right and 8 in the left atrium. The complete list of clinical cases is provided in Table 2. Data collection was performed according to the Helsinki Declaration guidelines on human research. The research protocol used in this study was reviewed and approved by the local review board. All patients provided written informed consent.

**Table 2:** Complete list of analyzed clinical AFL cases.

**No**.	**Atrium**	**Suspected mechanism**	**Description**
1	Right	Tricuspid valve reentry CCW	Typical right atrial flutter. Patient had previous PVI.
2	Right	Tricuspid valve reentry CCW	Typical right atrial flutter. Patient did not have previous PVI.
3	Left	Mitral valve reentry CW	Patient had previous PVI with anterior block line from mitral valve to left superior PV. A gap was detected in the block line.
4	Left	Mitral valve reentry CW	Patient had previous PVI. Posterior roof block from left superior PV to right superior PV.
5	Left	Mitral valve reentry CW	Patient had previous PVI. Gaps were found in the PVs from the previous ablation.
6	Left	Microreentry around PVI gaps	Patient had previous PVI. Gaps found in both left and right PVs from the previous ablation.
7	Left	Figure-of-eight reentry	Patient had previous PVI.
8	Left	Figure-of-eight reentry	Patient had previous PVI. Previously ablated with a FIRM system based on rotor detection.
9	Left	Figure-of-eight reentry	Patient had previous PVI. Gaps found in both left and right PVs from the previous ablation.
10	Left	Figure-of-eight reentry	Patient had previous PVI with anterior block line from mitral valve to left superior PV. Suspected figure-of-eight around right PVs and mitral valve. Gaps were detected in the right PVs and in the anterior block line.

Each AFL case was analyzed offline retrospectively, after the intervention, by directed network mapping, after exporting all EGMs and the corresponding 3D coordinates from the electro-anatomical mapping system. Only data collected before the actual ablation were analyzed.

For each case, the average directed network was created by following the steps reported in section 2.1. Briefly, *M* = 100 nodes were distributed over the mesh and Delaunay triangulation was performed to determine all sets of neighbors. Then, for each QRS complex, *N* = 5 different networks were created using a sliding window of length *l*, defined as half of the average distance between QRS complexes, with overlap of 100 ms. Given the time index *n*_*q*_ of the *q*-th QRS complex (i.e., R peak), the sliding window started at *n*_*q*_ − *l*/2 − 200 ms and was moved ahead 4 times with a stride of 100 ms. All networks were then “averaged” to obtain the average directed network. Once created, cycles were detected and then grouped together according to the algorithm described in section 2.2.

We finally compared the cycles found in the directed network with the clinical report of the patient. The clinicians provided information about the suspected AFL mechanism (as hypothesized after the successful ablation) and the corresponding part of the atrium involved (see Table 2). Depending on the suspected mechanism, we analyzed only the groups of cycles with a certain minimum number of nodes. In particular, in case of macroreentries, only cycles with at least 10 nodes were considered. On the other hand, for microreentry (case number 6), all cycles were analyzed. The evaluation of the correspondence between what was found by our algorithm and the clinical report was performed a-posteriori together with the clinician (the algorithm was run only once and before having any knowledge of the clinical reports; it was not adapted to the specific mechanism at hand).

## 3. Results

### 3.1. Validation in Sinus Rhythm

**Figure 2:**
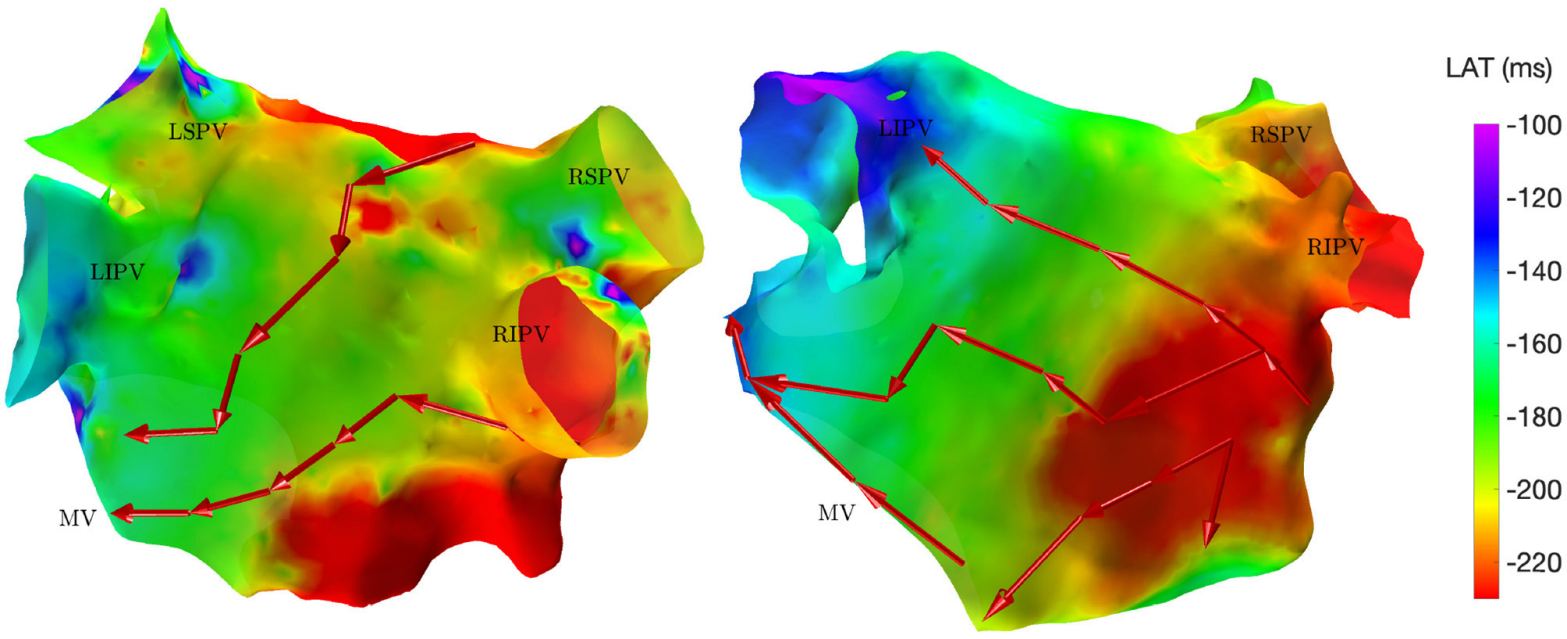
Electrical propagation pattern detected by directed network mapping (red arrows) superimposed over the LAT map (exported from CARTO® 3) for two subjects in sinus rhythm in posterior view. LSPV, left superior PV; LISP, left inferior PV; RSPV, right superior PV; RIPV, right inferior PV; MV, mitral valve.

The directed network mapping algorithm was first tested on the left atrium of the two patients in sinus rhythm and provided a result in line with the expectation. The electrical propagation started in the septum, propagated along both the anterior and posterior wall and ended around the left PVs. The LAT map provided by the CARTO® 3 system confirmed that the propagation found was properly detected. Figure 2 shows a few examples of the electrical propagation (red arrows) superimposed over the LAT map.

### 3.2. *In-silico* Validations

The efficacy of the directed network mapping was also tested in 6 different simulation settings, involving several types of AFL, induced on a 3D anatomical model of both atria. In each of the 6 setups, the algorithm was able to precisely detect the expected reentrant paths around the anatomical obstacles.

**Figure 3:**
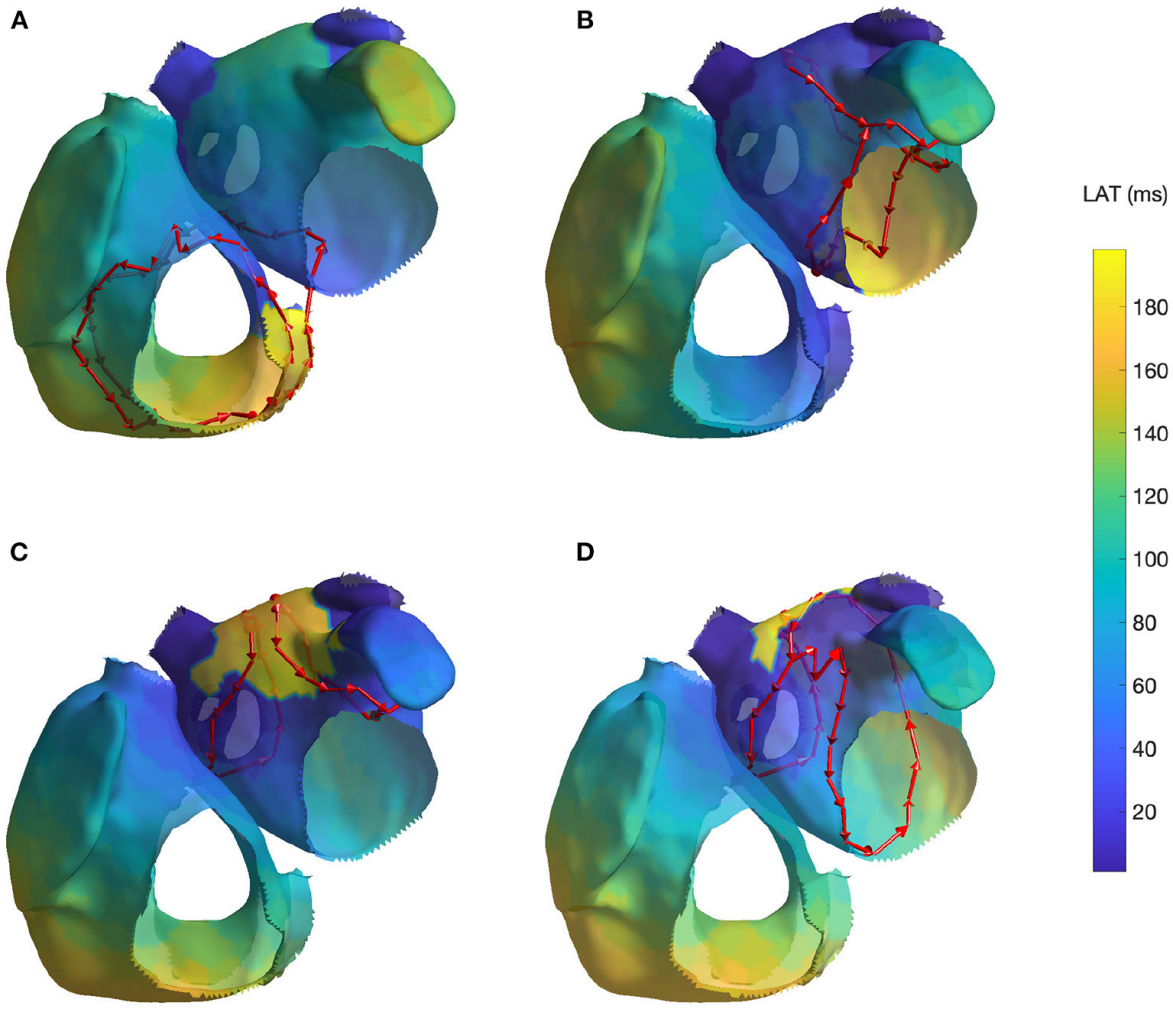
Cycles found on 4 AFL simulations: **(A)** Macroreentry on tricuspid valve CCW (simulation 1), **(B)** Macroreentry on mitral valve CW (simulation 3), **(C)** Figure-of-eight around both PVs in anterior direction (simulation 4) and **(D)** Figure-of-eight around right PVs in anterior direction (simulation 6; the threshold *t* was set to 0.45 for visualization purpose). Only one cycle randomly selected from each group has been plotted. The color map represents the time interval between the initial time of the stable phase of the simulation and the first atrial activation in each node.

For the reentry around the tricuspid valve with counterclockwise (CCW) direction (simulation 1), we detected three groups of cycles. The first one was the main driving cycle of a typical right AFL around the tricuspid valve. The second was the lower reentry going around the inferior vena cava. The third group was simply the combination of these two, sharing some of the nodes. In case of a tricuspid valve reentry with clockwise (CW) direction (simulation 2), the results were very similar, but only the first two groups were found. Figure 3A reports the cycles found for the first AFL simulation.

For the macroreentry around the mitral valve with CW direction (simulation 3), two cycles were detected by the algorithm. The first one was around the mitral valve, whereas the second was propagating around the left PVs in opposite direction. Figure 3B reports the cycles found for the third AFL simulation.

For the figure-of-eight simulation setting involving both PVs in anterior direction (simulation 4), two groups of cycles were found: one for each pair of PVs. On the other hand, for the posterior direction (simulation 5), four groups of cycles were found: two cycles with different radius for each pair of PVs. In both simulation scenarios, the identified cycles were matching what was induced in the simulation. Figure 3C reports the results for fourth AFL simulation.

In the final scenario of figure-of-eight (simulation 6), five groups of cycles were identified. These cycles were all describing the figure-of-eight pattern induced in the simulation by means of parallel cycles. Lowering the threshold to *t* = 0.45 produced two groups of cycles. For visualization purposes, Figure 3D reports the cycles found for the sixth AFL simulation with the lower threshold *t*.

The results of the simulated cases, not contained in Figure 3, are reported for completeness in the Supplementary Materials.

### 3.3. Validation With Clinical Data

A directed network was retrospectively created for each of the 10 EP studies recorded during ongoing AFL.

**Figure 4:**
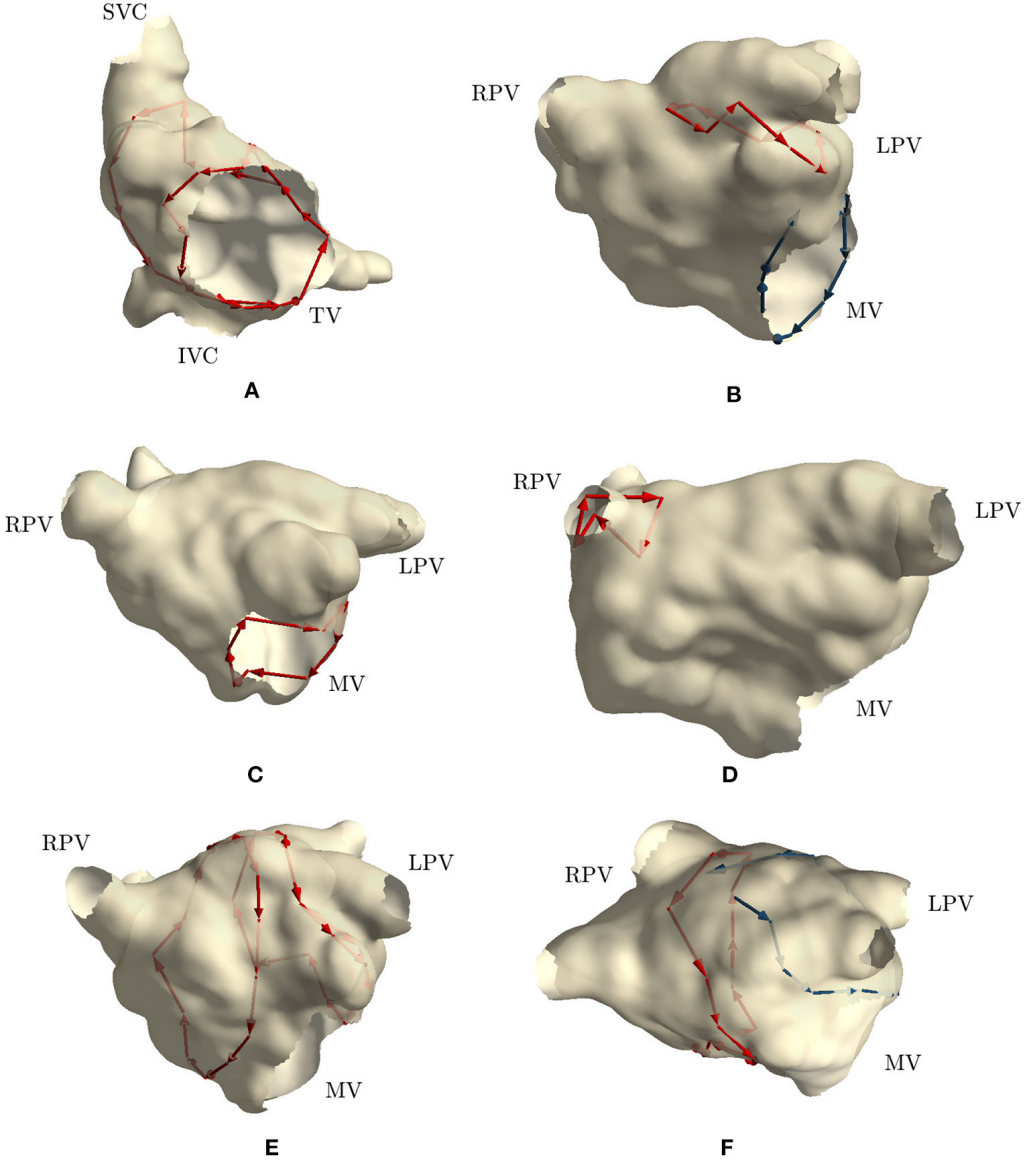
Cycles found on 6 clinical AFL cases: **(A)** Macroreentry around tricuspid valve CCW (case 2), **(B)** Macroreentry around mitral valve CW (case 3), **(C)** Macroreentry around mitral valve CW (case 4), **(D)** Microreentry through gap in PV ablation line (case 6), **(E)** Figure-of-eight around PVs in anterior direction (case 7), **(F)** and Figure-of-eight around PVs in anterior direction (case 8). Only one cycle randomly selected from each group has been plotted in red color. Blue arrows represent “broken” cycles that were manually traced with visual inspection. LPV, left PVs; RPV, right PVs; MV, mitral valve; TV, tricuspid valve.

The first two clinical cases presented tricuspid valve reentry with typical AFL (case 1 and 2). Two groups of cycles were identified for each case, similarly to those found in simulation 1 and 2, i.e., one cycle around the tricuspid valve and one around the inferior vena cava. These two cycles commonly appear together, and they are clinically treated by performing an ablation line at the floor of the right atrium between the inferior tricuspid annulus and the inferior vena cava (cavotricuspid isthmus). These two cases fully matched the expectations of the clinicians. Figure 4A reports the results of case 1.

Cases 3, 4, and 5 were left atrial reentries around the mitral valve in clockwise direction. In case 3, no cycle around the mitral valve was found, but one was detected around the left PVs in counterclockwise direction. Cycles with a lower number of nodes were not found either. We detected that three edges around the mitral valve were not built by the algorithm, while all other nodes were properly connected to form a cycle around the valve. Regarding the detected cycle, it commonly appears when mitral valve reentry is present. Similar results were found in simulation 3. Figure 4B reports the “broken” cycle in blue color and the identified one in red. In case 4, a cycle around the mitral valve was found but none was identified around the left PVs. This result was as expected. Indeed, the clinical report stated that a posterior roof block line from the left superior PV to the right superior PV was made in a previous ablation, which would prevent any rotation around the left PVs. Figure 4C shows the cycle identified for case 4. Finally, in case 5, no cycle resembling macroreentries was identified. Cycles of all lengths were thus plotted and small cycles around right PVs were found. These small cycles were in line with the gaps identified in the PVs and reported in the clinical report. Figure 4D reports the small cycles detected by the algorithm for this last case.

In case 6, as reported in the clinical report, during the EP study, a total of three gaps were found in the previous wide area circumferential ablation lines (WACA). Two gaps were found in the left WACA. On the left side, a WACA related, localized reentry mechanism involving both gaps could be detected. The right WACA line presented with a single posterior gap. The excitation wave-front was meandering with a reduced conduction velocity through the gap. The algorithm found three small cycles (less than 10 nodes) near the right inferior PV, while none was detected on the left side.

Cases 7, 8, 9, and 10 were suspected figure-of-eight macroreentries. In cases 7 and 8, the algorithm correctly detected the propagation pattern around the PVs. In particular, in case 7, two groups of cycles rotating around both left and right PVs were found, as reported in Figure 4E. On the other hand, in case 8, a complete cycle around the right PVs was found and a “broken” one, with only one edge missing, was identified on the left side. Figure 4F reports the results for this case. In case 9, no macroreentry was detected by the algorithm. However, when plotting cycles with fewer nodes, the algorithm found small cycles around the left and right superior PVs. These cycles might have been caused by gaps found on the PVs and reported in the clinical report. Finally, in case 10, the algorithm found only one group of cycles with at least 10 nodes. The cycles belonging to the group were found going around and through the right PVs and traveling along the anterior block line from a previous PVI. A gap in the right PVs was confirmed by the clinical report. However, this case was too complex to confirm the success of the algorithm.

To summarize, the algorithm (i) identified the exact same mechanism and its location in 4 cases (case 1, 2, 4, and 7); (ii) partially identified the mechanism in 4 cases (case 3, 6, 8, and 10); and (iii) failed to identify the mechanism in 2 cases (case 5 and 9).

The display of the cases, which did not enter Figure 4, are reported in the Supplementary Materials.

## 4. Discussion

In the present work, we verified that directed network mapping can be used to properly detect different types of AFL reentry. The method was applied to a broad range of simulations and clinical cases of AFL. First, we showed that directed network mapping can be used to accurately represent the electrical propagation pattern in sinus rhythm. Second, directed network mapping was able to correctly locate macroreentries in *in-silico* AFL models, where we tested 6 different scenarios. Finally, we tested the technique on 10 clinical cases of AFL and compared the results with clinical reports by expert electrophysiologists. Overall, in this retrospective pilot study, the algorithm proved to be satisfactory according to the clinicians' opinion with respect to the complex cases analyzed and has potential for clinical use. In fact, in addition to the fully identified cases (4/10), it is worth mentioning that the mechanisms of the cases labeled as “partially identified mechanisms” (4/10) were actually detected by the algorithm but not in their entirety. This makes it difficult to classify the performance of the algorithm with simply “identified” or “not identified”. Yet, we included in this group cases with even just a single edge missing (e.g., case 8—Supplementary Figure 14, Figure-of-eight reentry) or when the mechanism can be identified even just observing one cycle (e.g., case 8 and 10—Supplementary Figures 14, 16). For these cases, physicians may still get useful insights on the underlying mechanism and thus considering the goal as achieved (but we considered it as a partially identified mechanism instead of fully identified).

Over the years, network theory has had many different applications, but to the best of our knowledge, there was not a large amount of research in this domain to understand cardiac arrhythmias, until very recently. In the study by Vandersickel et al. (2019), directed networks were used to describe electrical excitation to extract the arrhythmia mechanism. In their work, the authors established a proof-of-concept using *in-silico* simulations of several activation patterns and clinical data of atrial tachycardia (AT) to demonstrate the wide applicability of directed networks in this domain. In their very recent follow-up study (Van Nieuwenhuyse et al., 2021b), authors evaluated the diagnostic accuracy of their method in more complex AT cases. They retrospectively analyzed 51 AT cases and compared the diagnoses made by their method with those of the experts based on high-density activation maps. They showed that cardiac mapping based on network theory could outperform high density activation mapping for specific types of AT (e.g., localized reentry), whereas for macroreentries, the directed network performed similarly to experts. These results hint that directed network may be a valuable tool during EP studies.

Our algorithm and the one proposed by Vandersickel et al. (2019) share the same goal, i.e., modeling the electrical propagation through directed networks and exploiting network theory to infer information about the arrhythmia mechanisms. Inspired by their study, we recently investigated the use of directed network mapping for AF characterization and rotor detection in computerized simulations (Vila et al., 2021). However, during AF, the creation of network based on LAT maps (as proposed by Vandersickel et al., 2019) become a difficult task. We then designed an algorithm directly estimating the time delay between activations recorded in nearby sites using a cross-correlation based algorithm. This approach was found to be more reliable with respect to computing the difference between LAT values in different sites (Shors et al., 1996; Cantwell et al., 2015). Motivated by these preliminary results, we started investigating on the applicability of directed networks for the identification of macroreentries in AFL. This problem required the implementations of a DFS algorithm for cycle detection, a grouping algorithm for cycles in similar locations, and a study specifically designed to test the algorithms on clinical cases: the work described in this manuscript. Very recently, a new contribution by the same research group has been released (Van Nieuwenhuyse et al., 2021b) and shares similarities with our analyses. First, both our work and theirs tested the capability of a directed network to correctly detect and group cycles. In this regard, our approach was based on a secondary graph taking into account the position of all nodes in the cycles, whereas they proposed a clustering technique based solely on the centroid of the cycles, that might group cycles with different orientations and with similar centers. Second, even though we both analyzed complex atrial arrhythmias, the objectives of the studies were different. Our study was focused on the validation of the algorithm, whereas they compared the output of the algorithm with the identification of the mechanisms performed by experts. Apart from different technical implementations and study designs, it was very satisfactory to find out that both studies corroborated the robustness of directed network and network theory to characterize AFL and its various mechanisms.

Other approaches to understand cardiac arrhythmias, based on network theory, are vastly distinct and mostly applied for AF characterization. For example, using high density contact mapping, directed networks were applied to describe AF by identifying recurring wavefront propagation patterns (Zeemering et al., 2013). AF was also described with a directed network by Richter et al. (2012), applying sparse modeling for the estimation of propagation patterns. Directed arrows can also be created based on the concept of Granger causality between different signals (Alcaine et al., 2017; Luengo et al., 2019). This approach could be an alternative way to generate the network, but it requires implementing a linear multivariate autoregressive model instead of deriving the activation times. Studies on undirected networks exist too. For example, Sun et al. (2014) created the network by quantifying the similarity between EGMs collected at different locations. Features from the network were then extracted and used to distinguish between SR and AF. In another study, Tao et al. (2017) used mutual information between pairs of nodes to build the network. Authors found that successful AF ablations led to networks with a higher connectivity with respect to unsuccessful ones. Overall, the use of directed network mapping, or in general network theory, to characterize the electrical propagation has several advantages with respect to a LAT map. In particular, it opens up to a whole new field of automated analyses. For example, the identification of reentries and focal points can be automatically and quickly performed, thus promoting a faster inspection of the ongoing arrhythmia.

We used unipolar EGMs as input to the directed network mapping. The major disadvantage of unipolar EGMs is that they also record substantial far-field ventricular signal, which interferes with the atrial activity. Because of the susceptibility to noise and far-field potentials, unipolar EGMs are not often used in clinical practice, leading to the routine use of bipolar EGMs (Stevenson and Soejima, 2005). However, bipolar EGMs can be sensitive to wavefront direction, bipole orientation, electrode size, interelectrode spacing, and the exact spatial location of the measurement is less precise (Ndrepepa et al., 1995; Nairn et al., 2020). Therefore, assessment of the exact LAT based on bipolar EGMs can introduce ambiguity, especially in low-voltage EGMs with multiple peaks (Haddad et al., 2014). To overcome the problem of far-field ventricular signal in unipolar EGMs, many different cancellation techniques have been developed. The classical approach is to employ the average beat subtraction (ABS) method, which considers the ventricular activity to be uncoupled from the atrial one. The method uses a single EGM, then calculates an average template of the ventricular activity (localized by means of the surface ECG), and subtracts it from the unipolar EGM, revealing the hidden actual atrial activity. Even though many variations to ABS have been proposed using the most diverse approaches, these methods still require signal acquisitions from several seconds to minutes, during which the catheter must be held still, and they are mostly applied offline. Signal acquisitions should be repeated after each change of catheter position, which prolongs the procedure to obtain state of the art map densities (more than 15,000 points on average; Takigawa et al., 2018), even if simultaneous wall contact of at least 20 electrodes per mapping position is assumed. For that reason, there is clearly a need to process the signals faster in real-time. During the last years some significant steps forward were taken in this direction with works such as Frisch et al. (2020) and Ríos-Muñoz et al. (2020). For example, Frisch et al. (2020) proposed to model the ventricular activity acquired at different locations during mapping and interpolate the voltage in locations not visited by the catheter. The main assumptions were that the ventricular activity varies smoothly across the atrial surface and that the atrial activity is not overlapped to the ventricular one (or, at least, uncorrelated). In this way, the model could be used afterwards to remove the ventricular activity.

The most common method to detect the existence of a cycle in directed networks is using DFS, by finding an edge that points to an ancestor of the current vertex (it contains a back edge). Since we are employing DFS and looking at all the vertices along with their edges, we have a runtime of *O*(*V* + *E*) with a space complexity of *O*(*V* + *E*) as well, where *V* is the number of nodes and *E* is the number of edges. The computational time required to build the directed network and to find the cycles was approximately 5 min computed on a Mac Book Pro- Intel core i9-9880H 3.3 GHz for a mapping phase lasting on average 20 min, without an optimized code. This method detects reentries very efficiently if such cycles are present in a network, but there were cases where some “broken” cycles existed, i.e., cycles of many nodes where only a few couples of edges were not created (Figure 4F reports one of such cases colored in blue). The cause for having those missing links might be the noise in the recordings or perhaps scarred or fibrotic tissue in that region. While in this work, when we suspected reentries in that specific anatomical region, we performed a close visual inspection, in the future the entire procedure should be revised designing a completely automatic algorithm, capable of coping with (a few) missing edges.

In this study, we investigated on directed network mapping for the identification of only AFL macroreentries. However, the technique showed some potential for microreentries as well. In fact, a few cycles of the smallest allowed dimension (i.e., 3 nodes) were detected for the clinical cases 5, 6 and 9 (see Supplementary Materials 11, 12, 15). The atrial area involving microreentries has been found to vary from few millimeters up to 2–3 cm as diameter. For example, Jaïs et al. (2009) reported evidence of slow conductive areas as small as 2 centimeter around which a reentry was observed. Furthermore, in 2020, Mantovan et al. (2020) were the first to report a microreentry circuit confined to a region size of a couple of millimeters. These areas range from 3 to 300 mm^2^. In our clinical cases, the average distance between nodes was 16.8 mm, that corresponded to a reentry with an area of approximately 120 mm^2^, hence the actual dimension of the smallest detected cycles was in line with what previously reported.

### 4.1. Limitations and Future Works

The current work is a proof of concept; still many different clinical settings are not yet tested. It remains to be seen how directed network mapping will characterize cardiac excitation in other types of arrhythmia, for example in cases with multiple rotors, or in a very fibrotic tissue. A possible limitation is that sequential mapping may fail to capture important dynamic changes in atrial electrophysiology when the arrhythmia is not stationary. In this proof-of-concept, we analyzed the entire mapping procedure, thus assuming AFL as stationary. This simplification might have led to spurious connections, possibly mitigated by the averaging network procedure put in place. Although the method does not require sequential mapping by itself, it is the predominantly available technology at present, and so we relied on that.

Other limitations regard the way the network was created. For example, the step involving the distribution of nodes on the mesh and Delaunay triangulation could create spurious connections between nodes that were not originally neighbors (for instance, in anatomical regions with high curvatures or narrow structures, e.g., appendage or PVs, see Figure 1B near the appendage). This problem will be mitigated in the future by downsampling the original mesh provided by the mapping system using dedicated algorithms. Moreover, we used the ventricular activity to temporally align EGMs after sequential mapping. Despite the coronary sinus is the most used time reference during EP studies for AFL, the use of ventricular activity should not affect substantially the creation of the network when AFL is stationary (i.e., fixed atrio-ventricular conduction ratio). We leave the comparison with the coronary sinus reference for future investigations. Furthermore, the number of simulated and clinical cases at disposal did not make feasible a systematic evaluation of the effects of the model parameters on the detected mechanism, hence we preferred to visually inspect the results. The influence of the parameters will be investigated on future works.

The current algorithm offers the visualization of the detected cycles as a means for guiding the ablation. For example, the algorithm may show that different cycles cross the same anatomical region (e.g., Figure 4A), thus making it as a suitable candidate spot for the ablation, due to the fact that both cycles will likely stop. However, the critical cycle or the ablation area still need to be delineated by the physician. It might not be excluded that, in the future, the identification of the critical cycle could be done automatically through the use of other technologies such as Signal Processing, Artificial Intelligence or High Resolution Computerized Simulations directly in the EP lab.

Another important extension of our method could be the conversion of the directed network into a weighted directed network by assigning a weight to each edge. The weight could be the level of “fractionation”, the conduction velocity, or other important features for the mechanism undergoing. This new approach requires a completely new set of algorithms where it is necessary to assign a physiological meaning on cycles built on edges with weights. However, this is not straightforward in our opinion, hence we leave this for future investigations. Yet, we believe that. together with an appropriate colormap, the visualization may become more informative and support better the planning of the optimal ablation.

An important step forward for the evaluation of the performance of our algorithm in AFL cycle detection would be the investigation on biatrial AFL and epicardial bridging. Biatrial AFL could be tackled by the presented algorithm if both the left and the right atrial geometry were given, connected and mapped. Since the septal connections could easily be considered for biatrial geometries, we are confident that the current implementation of our algorithm would perform well for biatrial AFL bridging via the septum. On the other hand, “invisible” bridges between the left and the right atrium that are not captured by the endocardial geometry acquired during mapping, alongside with epicardial bridges within one atrium, are more challenging. If the path taken by the AFL cycle is not part of the provided geometry, the present algorithm cannot identify it due to a lack of appropriate input data and may result in missing edges and interrupted cycles. Future works for cycle detection are in the directions of allowing tolerance in missing edges. Indeed, it is possible to upgrade the cycle-finding algorithm to incorporate properties of the atrial tissue in such a way to report the cycle even when a missing link (i.e., “invisible” bridge) is hampering the identification. This approach would likely be suitable for biatrial AFL and epicardial bridging but it requires extensive investigations.

A very significant potential application, and we believe the next step for directed network mapping, should be AF. AF is the most common sustained disorder of cardiac rhythm and is estimated to affect 1.5–2% of the general population with a prevalence that increases with age. Patients with AF have a five-fold higher risk of stroke and two-fold higher risk of death (Zoni-Berisso et al., 2014). Even though catheter ablation is at the forefront of the treatment of AF, it still produces moderate success rates (Verma et al., 2015), which is related to the lack of understanding of AF mechanisms. If the directed network mapping methodology may in the future offer some new insight in AF mechanisms remains to be seen. With respect to the application of network mapping in AF, so far we tested it in a simulation study, where we used a highly detailed computational 3D model of human atria in which sustained rotor activation was present (Vila et al., 2021). The main goal was to assess the potential of directed network mapping to characterize AF, and to use it for rotor detection. Additionally, in another recent study by Van Nieuwenhuyse et al. (2021a), the authors showed that network mapping can overcome some of the limitations of phase mapping, by being able to exclude the false rotors that phase mapping generates. The next important step in this direction is to analyze clinical AF cases.

## 5. Conclusion

Using network theory to characterize cardiac excitation represents an innovative and promising tool that has the potential to be used in an EP study for the treatment of AFL. In cases when the physician cannot unequivocally identify the driving mechanism using the LAT map and where several hypotheses can be formulated, directed network mapping could aid the operators by showing existing cycles in the network, possibly associated with conduction pathways sustaining AFL. In addition, this technology, along with dedicated visualization techniques, may represent an novel way to report the physicians an overall description of the arrhythmias in place, and speed up the planning of the ablation therapy.

## Data Availability Statement

The simulated dataset is available on request to the corresponding author. The clinical data related to this article will be shared with interested parties on reasonable request to the corresponding author and approval from the institutional review board.

## Ethics Statement

The studies involving human participants were reviewed and approved by Local Review Board of Medizinische Klinik IV, Städtisches Klinikum Karlsruhe, Karlsruhe, Germany. The patients/participants provided their written informed consent to participate in this study.

## Author Contributions

MV, MR, and RS designed the algorithm and performed the analyses. GL and ALo designed and performed the computerized simulations. LU, ALu, LG, and FL provided and curated the data of the clinical cases. LG and ALu provided interpretation of the results. All authors contributed to drafting and revising the manuscript.

## Funding

This work was funded by the European Union's Horizon 2020 research and Innovation programme under the Marie Skłodowska-Curie grant agreement No. 766082, MY-ATRIA (MultidisciplinarY training network for ATrial fibRillation monItoring, treAtment and progression) project. ALo, ALu, and LU gratefully acknowledge financial support by the Deutsche Forschungsgemeinschaft (DFG) through DO637/22-3, DO637/23-1, and LU 2294/1-1, respectively.

## Conflict of Interest

The authors declare that the research was conducted in the absence of any commercial or financial relationships that could be construed as a potential conflict of interest.

## Publisher's Note

All claims expressed in this article are solely those of the authors and do not necessarily represent those of their affiliated organizations, or those of the publisher, the editors and the reviewers. Any product that may be evaluated in this article, or claim that may be made by its manufacturer, is not guaranteed or endorsed by the publisher.
