# Supplementary material for: Atrial Flutter Mechanism Detection Using Directed Network Mapping
Source: Front Physiol. 2021 Oct 26;12:749635. doi: 10.3389/fphys.2021.749635 (PMC8577834; doi:10.3389/fphys.2021.749635)
Supplement: Supplementary file 1 [file Data_Sheet_1.PDF]

Supplementary Material

0.1 Figures

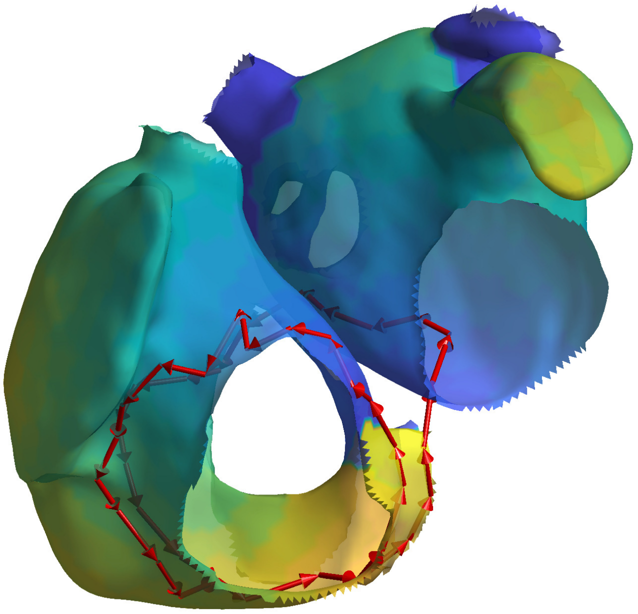

1

Figure S1. Simulation 1: Right atrium. Mechanism: Macroreentry around tricuspid valve in CCW direction.

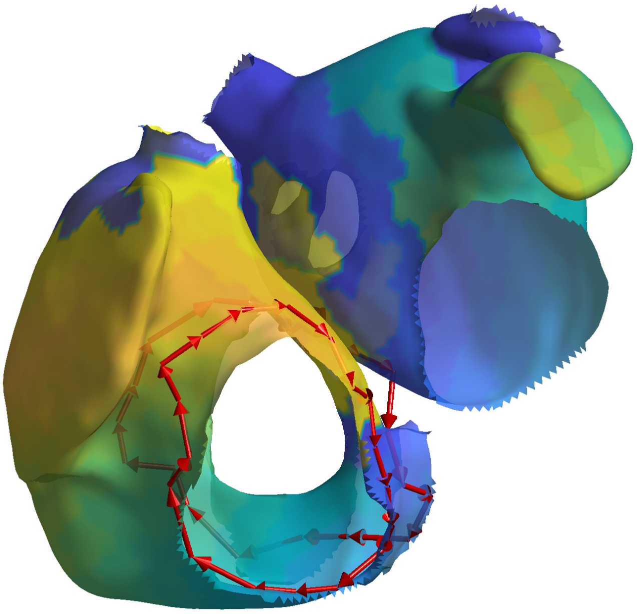

2

Figure S2. Simulation 2: Right atrium. Mechanism: Macroreentry around tricuspid valve in CW direction.

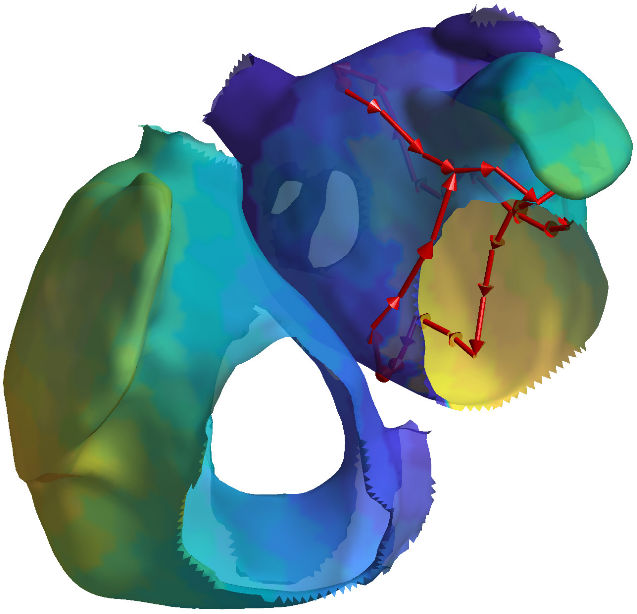

3

Figure S3. Simulation 3: Left atrium. Mechanism: Macroreentry around mitral valve in CW direction.

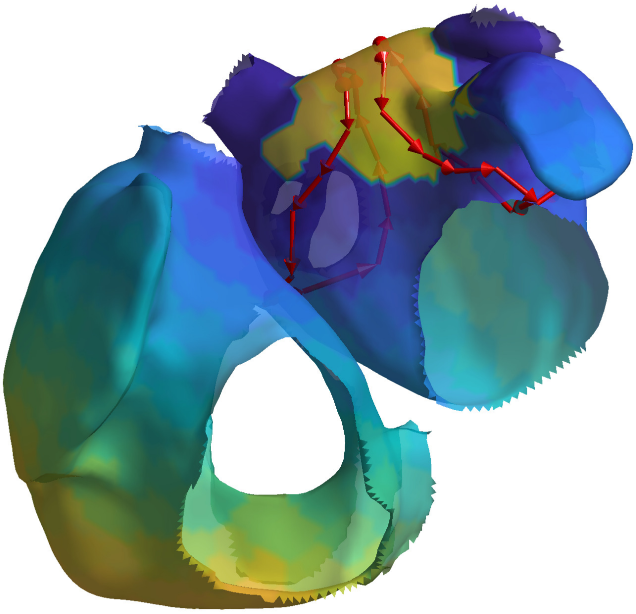

4

Figure S4. Simulation 4: Left atrium. Mechanism: Figure-of-eight macroreentry around both PVs in anterior direction.

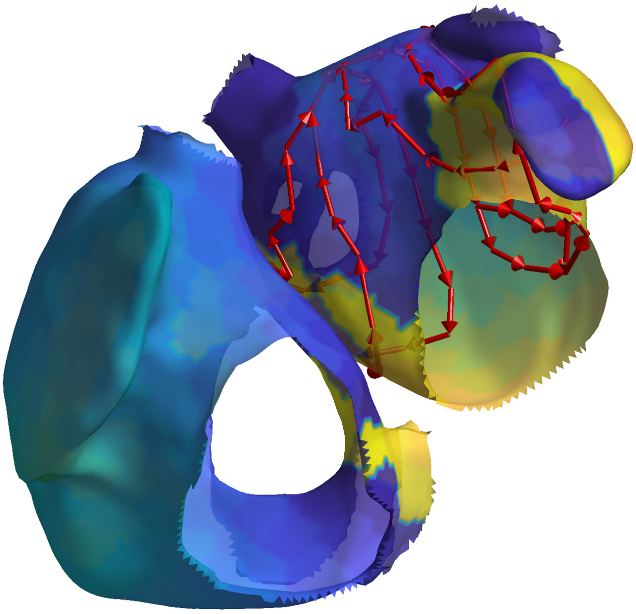

5

Figure S5. Simulation 5: Left atrium. Mechanism: Figure-of-eight macroreentry around both PVs in posterior direction.

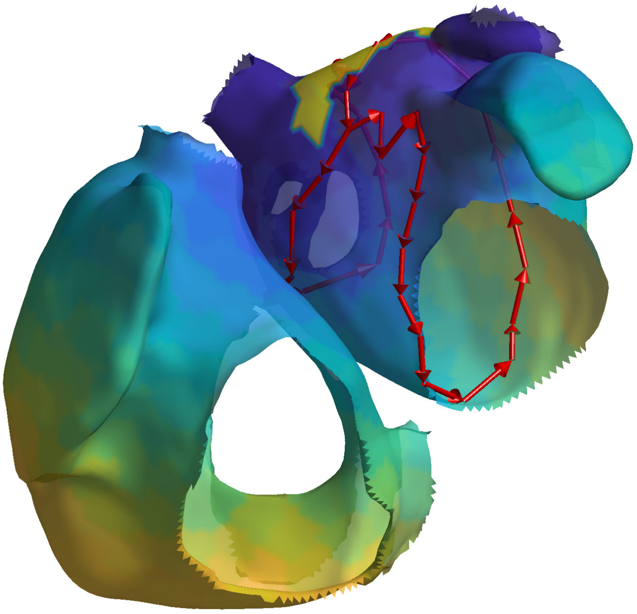

6

Figure S6. Simulation 6: Left atrium. Mechanism: Figure-of-eight macroreentry around right PVs in anterior direction.

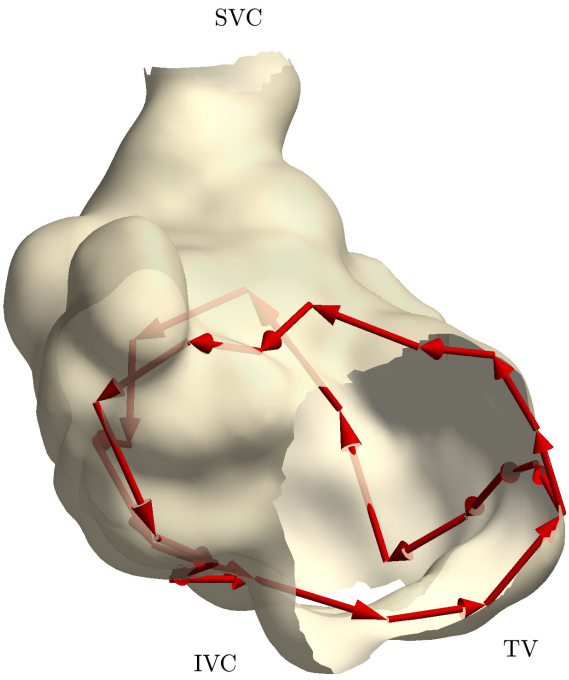

1

Figure S7. Case 1: Right atrium, Suspected mechanism: Tricuspid valve reentry CCW. Description: Typical right atrial flutter. Patient had previous PVI.

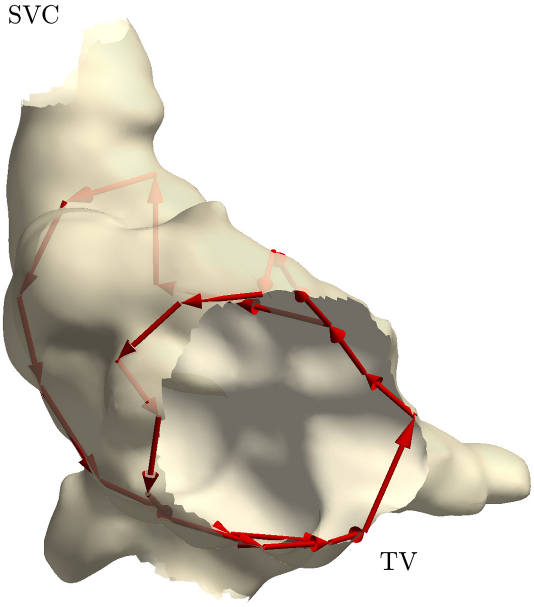

2

Figure S8. Case 2: Right atrium. Suspected mechanism: Tricuspid valve reentry CCW. Description: Typical right atrial flutter. Patient did not have previous PVI.

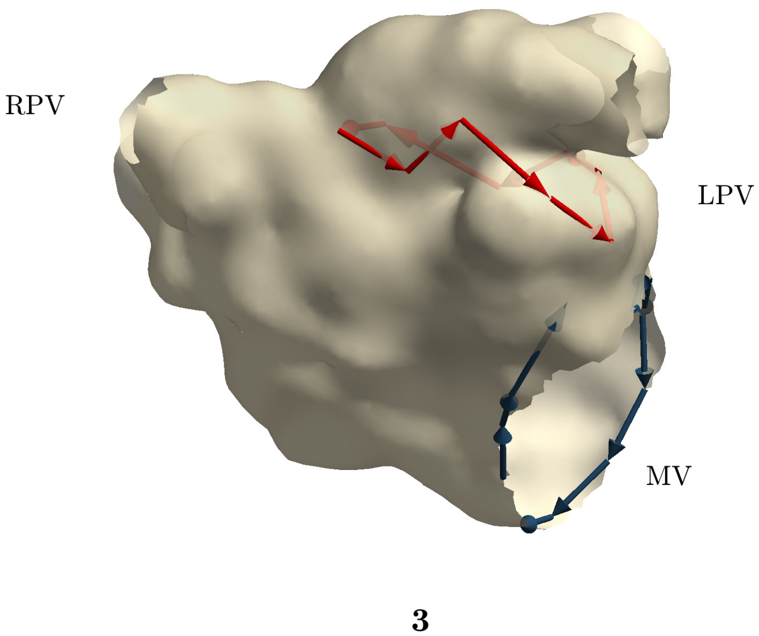

Figure S9. Case 3: Left atrium. Suspected mechanism: Mitral valve reentry CW. Description: Patient had previous PVI with anterior block line from mitral valve to left superior PV. A gap was detected in the block line.

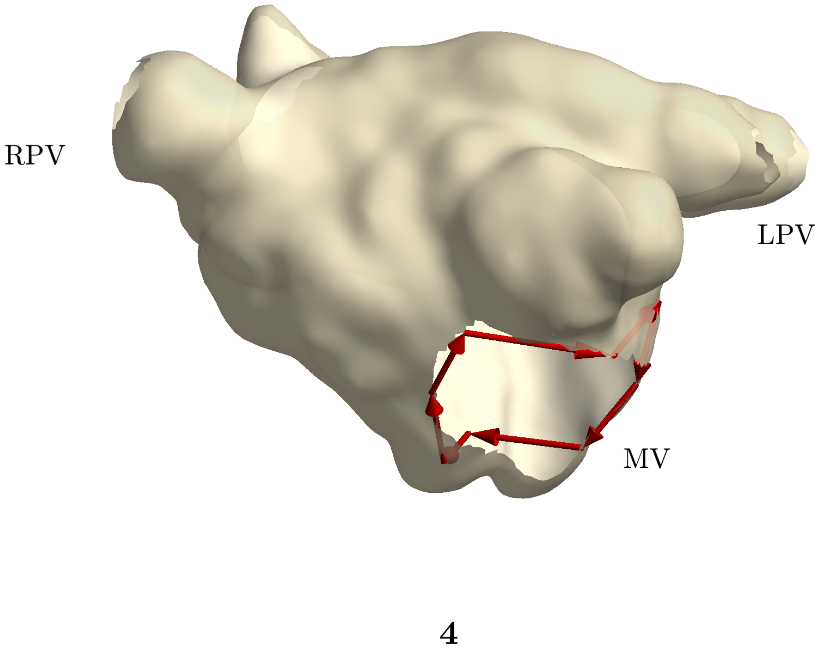

Figure S10. Case 4: Left atrium. Suspected mechanism: Mitral valve reentry CW. Description: Patient had previous PVI. Posterior roof block from left superior PV to right superior PV.

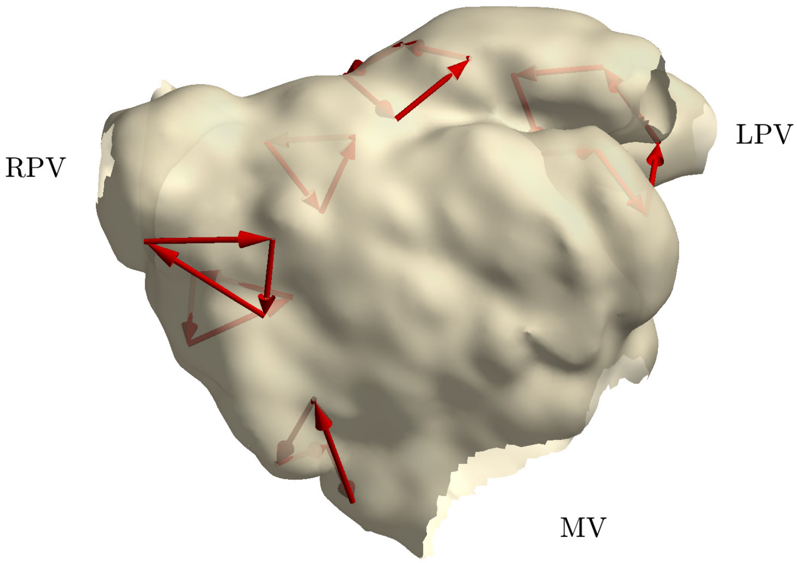

5

Figure S11. Case 5: Left atrium. Suspected mechanism: Mitral valve reentry CW. Description: Patient had previous PVI. Gaps were found in the PVs from the previous ablation.

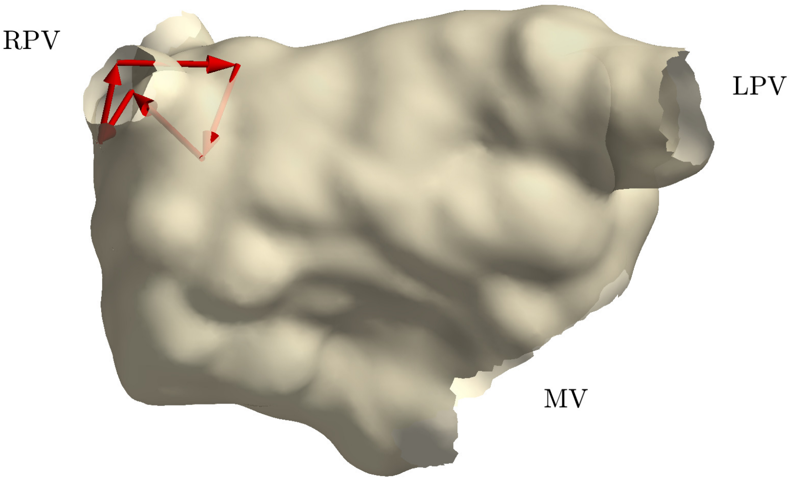

6

Figure S12. Case 6: Left atrium. Suspected mechanism: Microreentry around PVI gaps. Description: Patient had previous PVI. Gaps found in both left and right PVs from the previous ablation.

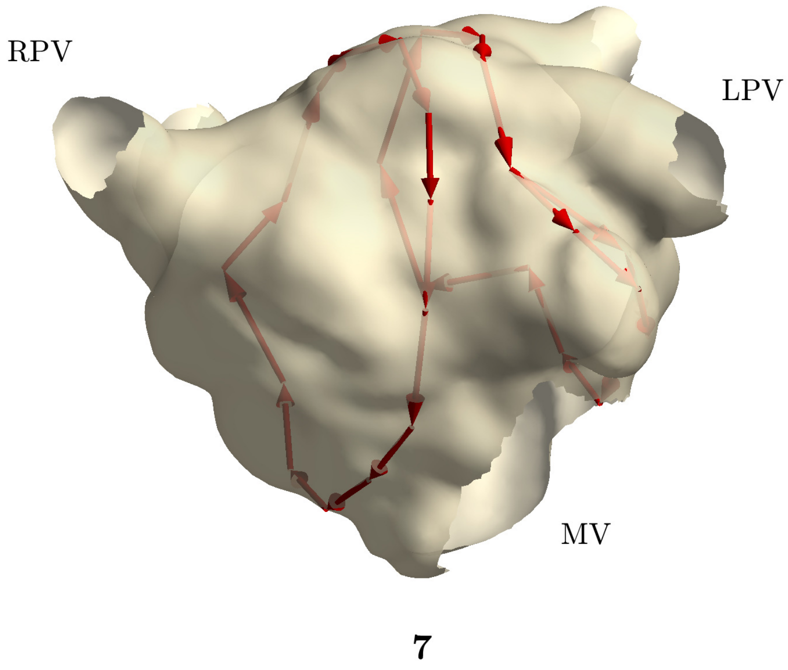

Figure S13. Case 7: Left atrium. Suspected mechanism: Figure-of-eight reentry. Description: Patient had previous PVI.

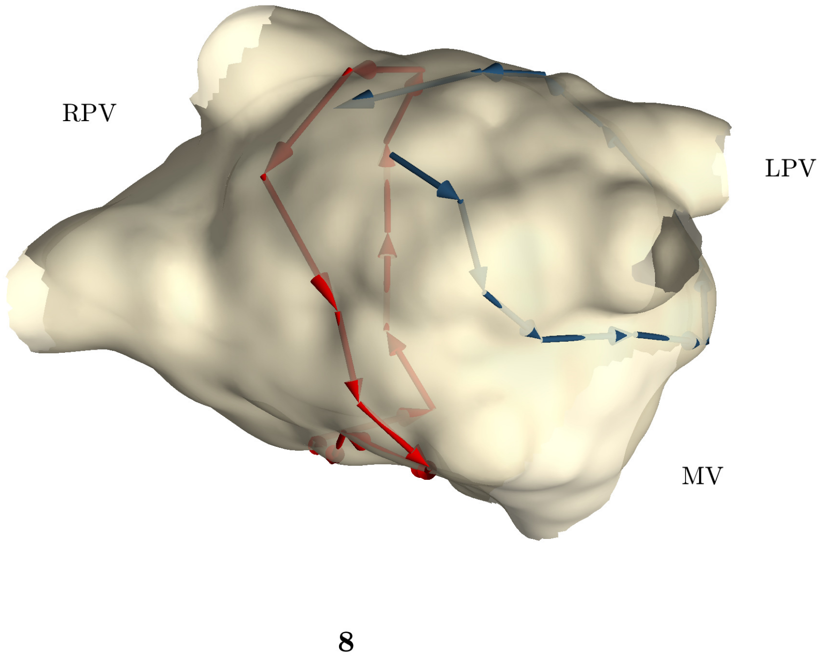

Figure S14. Case 8: Left atrium. Suspected mechanism: Figure-of-eight reentry. Description: Patient had previous PVI. Previously ablated with a FIRM system based on rotor detection.

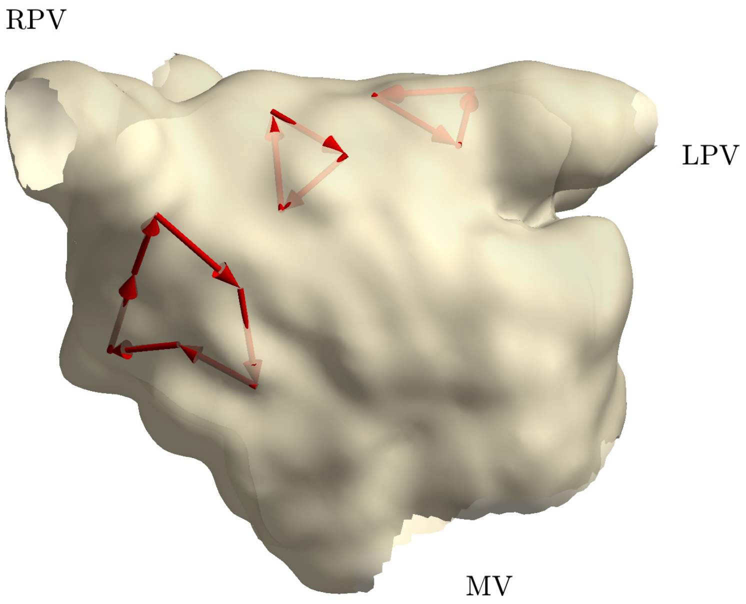

9

Figure S15. Case 9: Left atrium. Suspected mechanism: Figure-of-eight reentry. Description: Patient had previous PVI. Gaps found in both left and right PVs from the previous ablation.

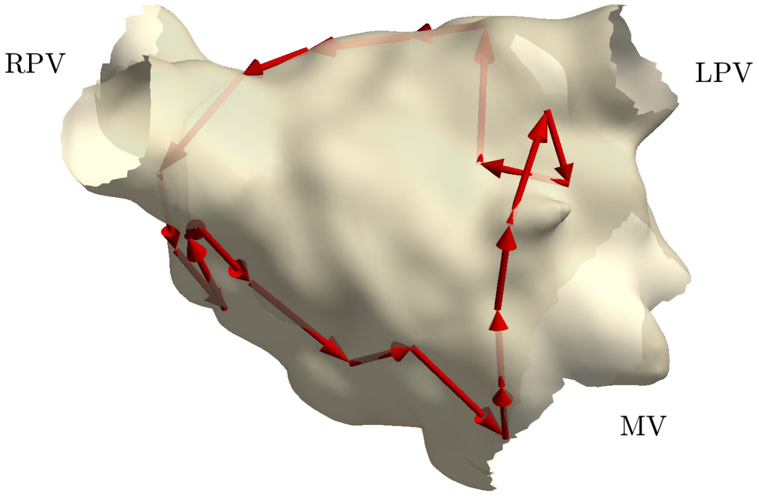

10

Figure S16. Case 10: Left atrium. Suspected mechanism: Figure-of-eight reentry. Description: Patient had previous PVI with anterior block line from mitral valve to left superior PV. Suspected figure-of-eight around right PVs and mitral valve. Gaps were detected in the right PVs and in the anterior block line.
